# Simple, mild, one-step labelling of proteins with gallium-68 using a tris(hydroxypyridinone) bifunctional chelator: a ^68^Ga-THP-scFv targeting the prostate-specific membrane antigen

**DOI:** 10.1186/s13550-017-0336-6

**Published:** 2017-10-25

**Authors:** Saima Nawaz, Gregory E. D. Mullen, Kavitha Sunassee, Jayanta Bordoloi, Philip J. Blower, James R. Ballinger

**Affiliations:** 1 Division of Imaging Sciences and Biomedical Engineering, King’s College London, St Thomas’ Hospital, London, UK; 2 Department of Nuclear Medicine, Guy’s and St Thomas’ Hospital, London, UK

**Keywords:** Tris(hydroxypyridinone) (THP), Prostate cancer (PCa), PSMA, Gallium-68 (^68^Ga), Single-chain antibody (scFv)

## Abstract

**Background:**

Labelling proteins with gallium-68 using bifunctional chelators is often problematic because of unsuitably harsh labelling conditions such as low pH or high temperature and may entail post-labelling purification. To determine whether tris(hydroxypyridinone) (THP) bifunctional chelators offer a potential solution to this problem, we have evaluated the labelling and biodistribution of a THP conjugate with a new single-chain antibody against the prostate-specific membrane antigen (PSMA), an attractive target for staging prostate cancer (PCa). A single-chain variable fragment (scFv) of J591, a monoclonal antibody that recognises an external epitope of PSMA, was prepared in order to achieve biokinetics matched to the half-life of gallium-68. The scFv, J591c-scFv, was engineered with a C-terminal cysteine.

**Results:**

J591c-scFv was produced in HEK293T cells and purified by size-exclusion chromatography. A maleimide THP derivative (THP-mal) was coupled site-specifically to the C-terminal cysteine residue. The THP-mal-J591c-scFv conjugate was labelled with ammonium acetate-buffered gallium-68 from a ^68^Ge/^68^Ga generator at room temperature and neutral pH. The labelled conjugate was evaluated in the PCa cell line DU145 and its PSMA-overexpressing variant in vitro and xenografted in SCID mice.

J591c-scFv was produced in yields of 4–6 mg/l culture supernatant and efficiently coupled with the THP-mal bifunctional chelator. Labelling yields > 95% were achieved at room temperature following incubation of 5 μg conjugate with gallium-68 for 5 min without post-labelling purification. ^68^Ga-THP-mal-J591c-scFv was stable in serum and showed selective binding to the DU145-PSMA cell line, allowing an IC50 value of 31.5 nM to be determined for unmodified J591c-scFv. Serial PET/CT imaging showed rapid, specific tumour uptake and clearance via renal elimination. Accumulation in DU145-PSMA xenografts at 90 min post-injection was 5.4 ± 0.5%ID/g compared with 0.5 ± 0.2%ID/g in DU145 tumours (*n* = 4).

**Conclusions:**

The bifunctional chelator THP-mal enabled simple, rapid, quantitative, one-step room temperature radiolabelling of a protein with gallium-68 at neutral pH without a need for post-labelling purification. The resultant gallium-68 complex shows high affinity for PSMA and favourable in vivo targeting properties in a xenograft model of PCa.

**Electronic supplementary material:**

The online version of this article (10.1186/s13550-017-0336-6) contains supplementary material, which is available to authorized users.

## Background

Prostate cancer (PCa) is the most common cancer in men in Europe, with 417,000 new cases diagnosed in 2012 [1] and 75,800 deaths predicted in 2016 [2]. Prognosis is very good if the disease is detected early when it is confined to the prostate gland. However, in the presence of metastatic disease, 5-year survival drops significantly [1]. There is a need for sensitive imaging techniques to assess metastatic and locally recurrent PCa in order to improve the outcome for these patients [3]. Prostate-specific membrane antigen (PSMA) is a well-established marker for PCa, with elevated expression in virtually all PCa but particularly in high-grade disease [4]. The radiopharmaceutical ^111^In capromab pendetide (ProstaScint, EUSA Pharma, licenced in the USA since 1997) is a monoclonal antibody (mAb) that targets an internal epitope of PSMA, which limits its utility because of poor accessibility to circulating mAb [5]. In contrast, the mAb J591 targets an external epitope and has shown more promise as an imaging agent, labelled with ^111^In for SPECT imaging, ^89^Zr for PET imaging and ^177^Lu for targeted radionuclide therapy; however, no commercial product has emerged yet [6–8].

Another route to targeting PSMA expression uses small molecule inhibitors of the enzyme *N*-acetylaspartylglutamate peptidase, a structural and functional homologue of PSMA [3]. Several small molecules labelled with technetium-99m, iodine-123, iodine-124 and iodine-131 have shown promise for SPECT or PET imaging or therapy of PCa [9–11]. More recently, gallium-68-labelled small molecules, such as PSMA-HBED-CC (also called DKFZ-PSMA-11), have been evaluated for PET imaging of PCa [12–15]. Gallium-68 is a positron emitter obtained by elution of a generator loaded with germanium-68. The 270-day half-life of germanium-68 allows the generator to be used for about 1 year. There are several commercial suppliers of ^68^Ge/^68^Ga generators, one of which is now licensed in some European countries, meaning that its eluate meets the specifications of the European Pharmacopoeia (*Ph Eur*). Gallium-68 forms complexes with appropriate chelators, thus in principle, opening the potential for preparation of PET radiotracers by kit procedures [15], analogous to ^99m^Tc in traditional radiopharmacy, rather than complicated radiochemistry and purification using cyclotron-produced fluorine-18.

**Fig. 1:**
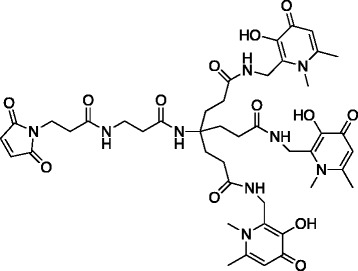
Chemical structure of the tris(hydroxypyridinone)-maleimide (THP-mal) chelator for gallium-68

The 68-min half-life of gallium-68 precludes its use with full-length mAbs because of their prolonged circulation times. Antibody fragments, particularly those of the scFv type, clear from circulation much more quickly [16]. To facilitate earlier imaging compatible with shorter half-life radionuclides such as gallium-68, our group has engineered fragments of the anti-PSMA mAb J591 for evaluation as imaging agents for PCa. We recently reported on a diabody radiolabelled with technetium-99m that showed specific binding to PSMA and favourable pharmacokinetics [17]. However, optimal images were not obtained until 4–8 h after injection, making it unsuitable for labelling with gallium-68. Accordingly, we have developed a single-chain variable region fragment (scFv) of J591 with an engineered C-terminal Cys residue (henceforth referred to as J591c-scFv) for conjugation to a bifunctional chelator (Fig. 1).

Protein labelling with gallium-68 is currently not straightforward, because currently available bifunctional chelators typically require relatively harsh labelling conditions such as high temperature, low pH and high chelator-conjugate concentration, often followed by a purification step [18–25]. These conditions are both inconvenient and often incompatible with protein-based radiopharmaceuticals. The aim of this work was to evaluate the use of tris(hydroxypyridinone) (THP), a high affinity chelator which efficiently binds gallium-68 rapidly at room temperature and neutral pH [15, 26–30], to avoid these problems and facilitate a mild, one-step protein labelling procedure without a need for a post-labelling purification step. By applying it in the context of the J591c-scFv antibody fragment, we also aimed to provide a preliminary preclinical evaluation of the radiopharmaceutical produced as a potential PET imaging agent for prostate cancer.

## Methods

### Antibody construction, expression and purification

As described previously [17], a single-chain variable region fragment (scFv) of J591 in the VH-VL orientation was PCR-amplified from the SFG P28z vector and cloned into pSEC-tag2 (Invitrogen, Carlsbad, CA, USA) mammalian expression vector pMS-C with N-terminal Ig-kappa leader and a C-terminal (His)_6_-tag followed by a Cys residue (J591c-scFv). HEK293T cells were transfected with the expression vector using Lipofectamine 2000 (Life Technologies, Paisley, UK), and transfected cells were selected with 100 μg/ml Zeocin (Life Technologies) before expanding to triple flasks for protein production. The J591c-scFv was purified from HEK293T culture supernatant by Ni-NTA chromatography (5 ml Ni-NTA Superflow Cartridge, Qiagen, Manchester, UK); the collected fractions were concentrated by centrifugal concentrations, then further purified by FPLC gel filtration using an AKTA system (Superdex 75 HR 10/30, GE Healthcare, Little Chalfont, UK). Purified protein in phosphate-buffered saline (PBS; pH 7.0) for subsequent maleimide conjugation was concentrated to > 1 mg/ml and stored in aliquots at − 80 °C. Purity was assessed by Coomassie staining after sodium dodecyl sulphate (SDS) polyacrylamide gel electrophoresis (SDS-PAGE; see Additional file 1) and analytical size-exclusion high-pressure liquid chromatography (HPLC) (BioSep SEC-s2000, Phenomenex, Cheshire, UK; see Additional file 1).

### Preparation of conjugate

The bifunctional chelator, THP-mal (referred to in some earlier publications as YM-103), was synthesised as described previously [26]. In order to couple the maleimide group of THP-mal, the J591c-scFv was reduced with a 30-fold molar excess of tris(2-carboxyethyl)phosphine (TCEP) to ensure maximum free Cys residue availability. THP-mal was added in 35-fold molar excess for 4 h at room temperature. THP-mal-J591c-scFv was purified by FPLC size-exclusion chromatography as described above. Proteins were analysed before and after TCEP reduction and after THP-mal conjugation by SDS polyacrylamide gel electrophoresis (as described above) and by electrospray mass spectrometry (Agilent 6500 Series Q-TOF LC/MS).

### Radiolabelling

A ^68^Ge/^68^Ga generator (IGG100, nominal 1.1 GBq, Eckert & Ziegler, Berlin) was eluted with 5 ml 0.1 N HCl (BioChemica, Sigma-Aldrich-Fluka). The eluate was trapped on a cation exchange cartridge (BondElut SCX, Varian) which had been preconditioned with 5.5 M HCl. The gallium-68 was eluted with 0.5 ml 5 M NaCl containing 12.5 μl 5.5 M HCl [21]. The eluate was neutralised with 1 M ammonium acetate. To optimise the required concentration of protein for efficient labelling, an aliquot of the neutralised eluate (12 MBq in 50 μl) was incubated with THP-mal-J591c-scFv at concentrations up to 1 μg/μl in a total reaction volume of 150 μl for 15 min at room temperature. To optimise the labelling incubation time, samples were taken from each incubation at times ranging from 10 s to 60 min. To determine the effect of temperature, 50 μl (25 μg protein) of THP-mal-J591c-scFv solution was incubated with 90 MBq gallium-68 in a total volume of 150 μl at 25 and 37 °C and samples were again taken at different time points (0, 5, 10, 20, 30, 40 and 60 min). In each optimisation, radiochemical purity was assessed by instant thin layer chromatography (ITLC) as detailed in Additional file 1. In all such experiments, unconjugated J591c-scFv (25 μg/50 μl) was also incubated with gallium-68 as a control. The labelling method arrived at after scaling up based on these optimisations, and subsequently used for in vitro and in vivo biological evaluation, was as follows: neutralised gallium-68-generator eluate prepared as described above (300 μl, 60–90 MBq) was added to THP-mal-J591c-scFv solution (80 μg, 200 μl). The resulting solution (700 μl) was incubated for 5 min at room temperature and then used without further processing for radio-HPLC (size exclusion), ITLC, serum stability, in vitro cell binding and in vivo experiments. Further details of radioanalytical methods are provided in Additional file 1.

### Serum stability studies

A volume of 100 μl of ^68^Ga-THP-mal-J591c-scFv, prepared as described above, was mixed with 100 μl saline and 200 μl fresh human serum and incubated at 37 °C. Under these conditions, approx. 0.57 nmol of protein containing 0.12 pmol of gallium-68 was present in the incubation. Samples were taken at 0, 30, 60, 120, 180, 240 and 300 min, immediately snap-frozen at − 80 °C and analysed together by ITLC to detect release of gallium-68 and by SDS-PAGE to discriminate serum protein-bound and antibody-bound activity. Gels were analysed by electronic autoradiography (Cyclone Plus, PerkinElmer, Waltham, USA) as described previously [17].

### Cell binding studies

The DU145 human PCa cell line was obtained from Cancer Research UK. A PSMA-overexpressing variant (DU145-PSMA) was produced as described previously [17]. The cell lines DU145 and DU145-PSMA were cultured in RPMI 1640 supplemented with 10% FBS, 2 mM l-glutamine and penicillin/streptomycin (cell culture reagents and consumables were purchased from PAA, Somerset, UK). Binding properties of ^68^Ga-THP-mal-J591c-scFv were analysed in homologous competition studies [17]. Cells (5 × 10^4^ DU145 or DU145-PSMA cells/well) were seeded in 96-well plates, grown overnight, then incubated with serial dilutions of J591c-scFv (7200 to 0.4 nM) and a constant concentration of ^68^Ga-THP-mal-J591c-scFv (2 nM; 0.33 MBq in 1 μl) at 4 °C for 40 min. Cells were washed with 3 × 100 μl cold PBS and lysed with 200 μl 0.5 M NaOH. Cell-associated activity was measured by gamma counting (1282 Compugamma, PerkinElmer, UK). Data were analysed with GraphPad Prism and fitted using a ‘one-site total binding’ algorithm to determine the IC50 of J591c-scFv with ^68^Ga-THP-mal-J591c-scFv as probe.

### PET imaging and biodistribution studies

Animal studies with male SCID beige mice aged ~ 8 weeks (Charles River, Margate, UK) were carried out in accordance with national and local regulations under UK Home Office project and personal licences. Cells (~ 3.5 × 10^6^ DU145 or DU145-PSMA cells) were injected subcutaneously on the flank in 90 μl of RPMI 1640 medium, and animals were used for imaging or ex vivo biodistribution studies after 25 days when tumours reached ~ 5 mm in diameter, as determined using a Vernier caliper. To determine the optimal time post-tracer injection at which to perform ex vivo biodistribution studies, dynamic positron emission tomography was performed with a small-animal PET/CT scanner (NanoPET, Mediso, Budapest, Hungary) [31] using isoflurane anaesthesia and respiratory monitoring, for 3 h after tail vein injection of ^68^Ga-THP-mal-J591c-scFv (7 MBq, 10 μg protein, 90 μl) in one mouse with each tumour type, with CT images acquired after each PET scan. PET images were reconstructed with Nucline software (Bioscan, Washington, DC, USA). Data extraction from PET images is described in Additional file 1. For ex vivo biodistribution studies, ^68^Ga-THP-mal-J591c-scFv (7 MBq, 10 μg protein, 90 μl) was injected via the tail vein of mice bearing either DU145 or DU145-PSMA xenografts, and mice were sacrificed after 90 min (four mice/group). Organs were dissected, briefly washed in PBS, blotted dry and weighed. Activity in whole organs and tumours was measured by gamma counting and is expressed as percent injected dose per gram of tissue (%ID/g).

## Results

### Antibody construction, expression and purification

J591c-scFv was produced in HEK293T cells with yields of purified protein (purity > 95%) of 4–6 mg/l culture supernatant. On SDS-PAGE analysis, J591c-scFv showed a molecular weight of ~ 30 kDa with covalent or non-covalent dimers evident at ~ 60 kDa (Additional file 1: Figures S1 and S2). Although gel electrophoresis analysis was consistent with the presence of both monomeric J591c-scFv and its disulfide-linked dimer (reducible to the monomer with TCEP, see below), the deconvoluted electrospray mass spectrum (Additional file 1: Table S1 and Figure S1) before TCEP treatment showed no evidence of dimeric protein and also that the monomeric protein (27923) was 119 Da heavier than predicted from its amino acid sequence (27804), corresponding to a conjugate formed by a disulfide bond between the terminal Cys residue and a non-peptide bound cysteine; only minor peaks were detected corresponding to reduced J591c-scFv (27804). Following reduction of dimers with TCEP (see Additional file 1: Figure S2), the mass spectrum (Additional file 1: Table S1 and Figure S1) showed the presence of reduced J591c-scFv (27804, consistent with the reductive removal of the disulfide-linked cysteine) and trace amounts of dimer (55606-55607; the intensity of this envelope was too small to determine whether this was disulfide-linked or non-covalent dimer).

### Preparation of conjugate

After reduction with TCEP, the J591c-scFv could be coupled with the maleimide-containing bifunctional chelator THP-mal and purified by size-exclusion chromatography (see Additional file 1: Figure S3). High-resolution mass spectrometry of the product showed one major peak with the molecular weight (28724) expected for the THP-mal-J591c-scFv conjugate as well as residual unmodified protein (Additional file 1: Table S1 and Figure S1), together with trace amounts of unconjugated non-covalent or disulfide-linked dimer (55607.7) as observed for the unconjugated protein. Size-exclusion FPLC purification (Additional file 1: Figure S3) showed a major protein species present after the conjugation, eluting at 10 min, with a small amount of larger protein eluting at 8 min corresponding to disulfide-linked or non-covalent dimer. SDS-PAGE after removal of excess THP-mal by size-exclusion chromatography showed blue-stained bands around 30 and 60 KDa consistent with the presence of both monomeric and dimeric protein (Additional file 1: Figure S5, lane 3). Size-exclusion HPLC of this purified protein showed peaks eluting at ca. 8.5 min and its non-covalent or disulfide-bonded dimer eluting at 7.8 min, together with a small amount of unknown high-molecular weight impurity eluting at 5.5 min (Additional file 1: Figure S5).

### Radiolabelling

**Fig. 2:**
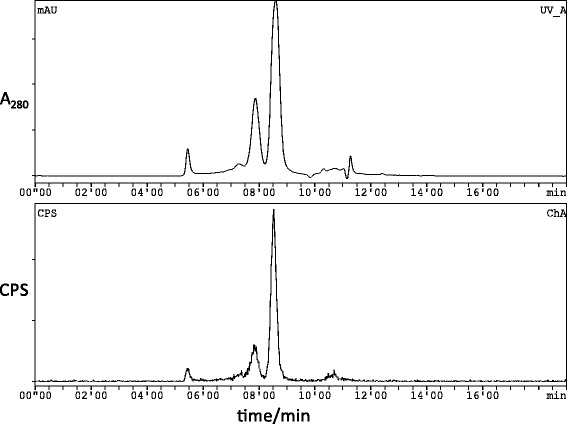
Size-exclusion HPLC of THP-mal-J591-scFv after radiolabelling with gallium-68 (SEC-2000, column, flow rate 1 ml/min, UV detection at 280 nm). Top: UV chromatogram, showing THP-mal-J591-scFv eluting at ca. 8.5 min and its non-covalent or disulfide-bonded dimer eluting at 7.8 min; bottom: simultaneously recorded radiochromatogram, showing radioactivity associated with THP-J591c-scFv (8.5 min, 84%), dimer (7.8 min, 14%) and unbound gallium-68 (10–12 min, < 2.6%). A small radioactive higher molecular weight protein was also observed (5.5 min, < 2.5%)

THP-mal-J591c-scFv could be labelled with ^98^Ga acetate quantitatively within 5 min at room temperature and neutral pH at concentrations ≥ 0.25 μg/μl (total 5 μg protein). The radiolabelling was evaluated by ITLC, showing that non-protein-bound gallium-68 was below the detection limit of 1%. Size-exclusion radiochromatography (Fig. 2) showed less than 2.6% free gallium-68 and > 97% of radioactivity in the form of labelled protein comprising ^68^Ga-THP-mal-J591c-scFv (81.0%, 8.5 min), a radiolabelled dimeric form (14.3%, 7.8 min, also detected by gel electrophoresis) and an unknown high molecular weight impurity (< 2.1%). Thus, radiochemical yields (decay corrected) of at least 97% were attained within an incubation time of 5 min (< 10 min post-generator elution) at room temperature. Unconjugated J591c-scFv showed no labelling by these methods. A series of optimisation experiments (see Additional file 1) showed that increasing temperature to 37 °C did not improve radiochemical yield at 5 min. At 25 °C with concentrations above 0.25 μg/μl, labelling was complete in the samples taken at 10 s (although the labelling reaction may have continued during the drying time of the ITLC spot) but with protein conjugate concentrations below 0.2 μg/μl, longer incubation times were required to achieve acceptable purity. For subsequent in vitro and in vivo characterisation of the labelled product, 5–80-μg batches were radiolabelled.

### Serum stability studies

^68^Ga-THP-mal-J591c-scFv was stable, showing no change in speciation of gallium-68 when incubated in human serum at 37 °C for 6 h with no evidence of loss of free gallium-68 or transchelation to serum proteins (Additional file 1: Figure S5).

### Cell binding studies

**Fig. 3:**
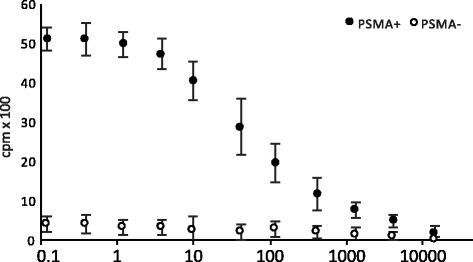
Binding of ^68^Ga-THP-mal-J591c-scFv to DU145 parental and DU145-PSMA cell lines in the presence of increasing quantities of J591c-scFv. Each data point is a mean of 3 measurements ± SD

^68^Ga-THP-mal-J591c-scFv showed low binding to parental DU145 cells, which do not express PSMA (Fig. 3). In contrast, ^68^Ga-THP-mal-J591c-scFv showed binding to DU145-PSMA cells that was > 10-fold higher and dependant on the J591-scFv concentration. Using ^68^Ga-THP-mal-J591c-scFv as a probe for binding to DU145-PSMA cells over a range of concentrations of unconjugated J591c-scFv, we obtained an IC50 value of 31.5 nM for J591c-scFv.

### Xenograft studies

**Fig. 4:**
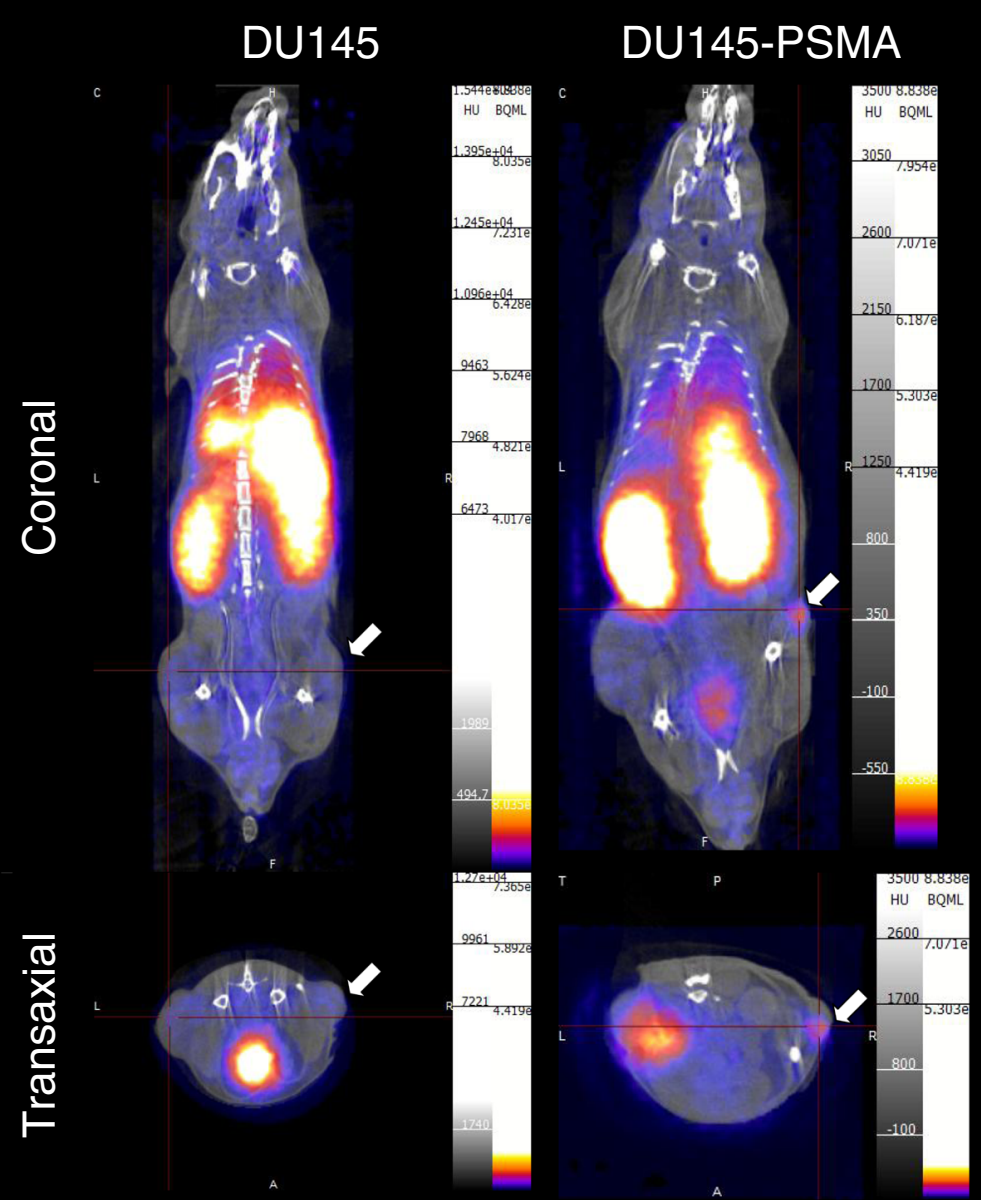
Coronal and transaxial images of mice bearing DU145 parental or DU145-PSMA xenografts using summed data acquired 60–90 min post-injection of 7 MBq (10 μg) ^68^Ga-THP-mal-J591c-scFv. Arrows mark location of xenograft tumour

To determine the appropriate time for ex vivo biodistribution study, mice bearing DU145 or DU145-PSMA xenografts were injected with ^68^Ga-THP-mal-J591c-scFv and assessed by dynamic PET/CT imaging over 3 h. Rapid distribution and clearance from circulation of ^68^Ga-THP-mal-J591c-scFv was observed, with extensive accumulation in the kidneys, urinary bladder and liver, all reaching a plateau by 60 min. The DU145 (PSMA negative) xenograft showed no significant uptake above the surrounding background (Fig. 4). In contrast, activity in the DU145-PSMA tumours was visible and reached a plateau after 60 min (Additional file 1: Figure S6).

**Fig. 5:**
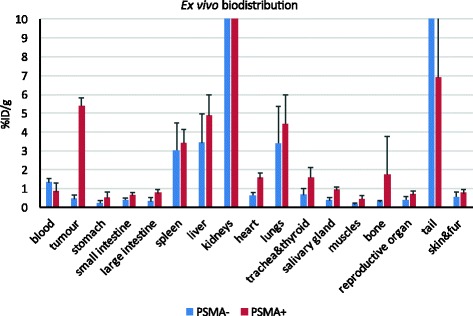
Ex vivo biodistribution (%ID/g) of ^68^Ga-THP-mal-J591c-scFv in SCID mice bearing DU145 parental or DU145-PSMA xenografts 60–90 min post-injection. Each bar represents a mean of 4 mice + SD

Based on the time course observed on imaging, ex vivo biodistribution studies were carried out at 90 min. As seen in Fig. 5 and Additional file 1: Table S2, accumulation of ^68^Ga-THP-mal-J591c-scFv in the DU145-PSMA tumour was 5.4 ± 0.5%ID/g, while the DU145 tumour contained 0.5 ± 0.2%ID/g (mean ± SD, *n* = 4). The tumour to blood ratios were 6.8 ± 0.6 and 0.4 ± 0.2 (*t* test, *P* < 0.0004), while the tumour to muscle ratios were 13.5 ± 3.1 and 2.5 ± 0.5 (*P* < 0.004), for DU145-PSMA and DU145 tumours, respectively. The weights of the DU145-PSMA and DU145 tumours were not significantly different (0.30 ± 0.08 g and 0.24 ± 0.17 g, respectively). The kidneys, liver and spleen were the only other organs containing significant levels of gallium-68.

## Discussion

J591c-scFv was engineered with a free Cys residue to allow coupling with THP-mal, a maleimide-containing bifunctional version of the tripodal chelator THP that labels at room temperature and neutral pH [15, 26–30]. The protein was obtained from the supernatant of a transfected HEK293T cell suspension in a purified yield of 4–6 mg/l. Gel electrophoresis and electrospray mass spectrometry showed that the protein was present mainly in oxidised form, with a disulfide bond to a Cys from the medium. This species reverted to the reduced J591c-scFv upon treatment with TCEP and could then be efficiently conjugated to THP-mal to give a single protein species as judged by gel electrophoresis and size-exclusion HPLC. After the removal of TCEP by size-exclusion chromatography, however, subsequent handling resulted in the formation of variable amounts of a covalent or non-covalent dimeric form detectable by size-exclusion HPLC, PAGE and mass spectrometry.

Although the eluate of the current ^68^Ge/^68^Ga generator meets the specification of the *Ph Eur* and thus is suitable for administration to patients, and THP conjugates can be labelled efficiently using unprocessed eluate [15], the present work was performed before the latter discovery and utilised a preconcentration step in which gallium-68 was trapped on a cation exchange cartridge and eluted in a small volume of NaCl/HCl [21]. The eluate was neutralised with ammonium acetate and incubated with THP-mal-J591c-scFv at room temperature. After surveying a range of labelling conditions, protocols were established involving 5-min incubation at room temperature of aliquots of 5–80 μg protein at concentrations of 0.25–0.4 μg/μl. This extremely simple and quick protocol gave quantitative labelling and required no post-labelling purification or harsh conditions such as low pH or elevated temperature that might denature the protein, and is a suitable basis for future development of a simple, one-step kit-based GMP labelling procedure for proteins. This confirms the advantages, in the context of protein labelling, of the THP chelator over other potential chelating systems that require either heat or low pH, or both, to achieve efficient labelling.

After radiolabelling, small and variable amounts of a heavier labelled protein were detectable by size-exclusion radiochromatography (Fig. 2) and by radio-gel electrophoresis (Additional file 1: Figure S5, lane 3). The failure of electrospray mass spectrometry to detect this higher molecular weight species suggests that it is a non-covalent dimer that readily dissociates even under non-reducing conditions. The product was therefore allowed to proceed to biological evaluation.

^68^Ga-THP-mal-J591c-scFv was shown by gel electrophoresis to be stable in human serum against transchelation by serum proteins (notably transferrin as previously established [26]) for periods longer than likely PET scanning times for gallium-68 tracers. A competitive binding study using DU145-PSMA cells (with non-PSMA-expressing DU145 cells as a control), and tracer level ^68^Ga-THP-mal-J591-scFv as a probe, gave an IC50 value of 31.5 nM for J591c-scFv, confirming that J591c-scFv has a suitable affinity for PSMA to serve as the basis of a PSMA in vivo imaging agent and that the labelled conjugate ^68^Ga-THP-mal-J591c-scFv shows specific, saturable binding to the PSMA-expressing cells.

When injected intravenously into SCID mice and imaged over 4 h by small-animal PET/CT, ^68^Ga-THP-mal-J591c-scFv showed rapid clearance from the body via urinary excretion. Highest levels were retained in the kidneys and significant but lower levels in the liver and spleen. Accumulation in the DU145-PSMA variant was visible on the scans and was at least 10-fold higher (5.4%ID/g) than that in the non-PSMA-expressing tumour. In both types of tumour, peak accumulation occurred 60–90 min after injection. The high, PSMA-dependant tumour uptake and the early attainment of high tumour to background ratio suggest that ^68^Ga-THP-mal-J591c-scFv can be used as a tracer for PET imaging of PSMA expression in PCa. Despite binding to PSMA by a different mechanism, when measured in the same cell line (DU145-PMSA), the affinity of ^68^Ga-THP-mal-J591c-scFv for PSMA is comparable to that of ^68^Ga-HBED-CC-PSMA and ^68^Ga-DOTA-PSMA [15], which are both examples of the new generation of small molecule-based tracers, used clinically for prostate cancer imaging, and which bind to the enzymatically active peptidase site of PSMA [3, 14]. Its in vivo uptake in the DU145-PSMA tumour and kidneys is similar to that of ^68^Ga-PSMA-HBED-CC and ^68^Ga-THP-PSMA in the same tumour model [15]. However, its uptake in the liver is much higher, and overall, it has no advantage as an alternative to these tracers.

## Conclusions

We have shown that the THP bifunctional chelator is potentially a solution to the problem that labelling of proteins with gallium-68 typically entails harsh conditions such as high temperature and acidic pH, as well as post-labelling purification, thus opening the door to kit-based protein labelling with gallium-68. We have produced an scFv variant of the anti-PSMA mAb J591 derivatised with THP that is amenable to simple, rapid, quantitative, one-step room temperature labelling with gallium-68 at neutral pH. The resultant gallium-68 complex shows high affinity for PSMA and can detect PSMA-expressing tumours by PET imaging in vivo.

## Additional files

Additional file 1: Table S1. Deconvoluted electrospray mass spectra of J591c-scFv pre- and post-reduction and conjugation with THP-mal. **Table S2.** Ex vivo biodistribution data (%ID/g) for ^68^Ga-THP-mal-J591-scFv in mice bearing DU145 (PSMA-) and Du145-PSMA (PSMA+) (mean and standard deviation are shown; *n* = 4 in each group). **Figure S1.** Electrospray mass spectra of J591c-scFv pre-TCEP treatment (top, 27,923.2 corresponds to disulfide formed from J591c linked to cysteine via a disulfide bond), J591c-scFv post-TCEP treatment (middle, 27,803.9 corresponds to J591-scFv), and TCEP-treated J591-scFv after incubation with THP-mal (bottom, 28,723.7 corresponds to THP-mal-J591-scFv adduct). **Figure S2.** The effect of different molar excesses of TCEP on dimerisation of J591-scFv. Lanes: 1: molecular weight markers; 2: non-reduced protein; 3: reduced protein (NuPAGE reducing agent); 4: 0.4:1 M ratio TCEP to protein; 5: 1:1 M ratio; 6: 2:1 M ratio; 7: 5:1 M ratio; 8: 10:1 M ratio; 9: 15:1 M ratio; 10: 20:1 M ratio; 11: 30:1 M ratio. **Figure S3.** FPLC purification of THP-mal-J591-scFv conjugate on Superdex 75 HR 10/30 size-exclusion column eluted with phosphate-buffered saline at 0.5 ml/min. Conjugate elutes before free THP-mal. Free THP-mal can be seen eluting at 15–18 min, monomeric THP-conjugated protein at 10 min and dimeric protein at 8 min. **Figure S4.** Exemplar data showing radiolabelling efficiency (% protein bound, determined by ITLC) at different protein conjugate concentrations and times. At concentrations of 0.25 mg/ml or higher, labelling efficiency was 100% at all time points from 10 s onward (the first data point in each series represents a sample taken after 10 s incubation). **Figure S5.** SDS-PAGE analysis of serum stability of ^68^Ga-THP-mal-J591-scFv. A: radioactive gel analysed with cyclone phosphor imager. B: gel stained with Coomassie blue. Lanes: 1: molecular weight markers; 2: human serum incubated with ammonium acetate-buffered ^68^Ga eluate; 3: radiolabelled ^68^Ga-THP-mal-J591-scFv conjugate control (without serum incubation); 4: conjugate incubated with human serum for 1 min; 5: 30 min; 6: 60 min; 7: 120 min; 8: 180 min; 9: 240 min; 10: 360 min. **Figure S6.** Time course of ^68^Ga-THP-mal-J591c-scFv activity (SUVmax) in DU145-PSMA xenografts derived from serial images in a single mouse determined by PET imaging. A region of interest near the centre of each organ was drawn from which SUVmax was determined in each 15 min bin. **Figure S7.** Ex vivo biodistribution data for ^68^Ga-THP-mal-J591-scFv in mice bearing DU145 (PSMA-, blue) and DU145-PSMA (PSMA+, red), 90 min post-injection. Error bars represent standard deviation (*n* = 4 per group). Data are the same as those shown in the main manuscript (Fig. 5) but expanded to include kidneys. (PDF 1307 kb)
