# Supplementary material for: Simple, mild, one-step labelling of proteins with gallium-68 using a tris(hydroxypyridinone) bifunctional chelator: a 68Ga-THP-scFv targeting the prostate-specific membrane antigen
Source: EJNMMI Res. 2017 Oct 25;7:86. doi: 10.1186/s13550-017-0336-6 (PMC5655379; doi:10.1186/s13550-017-0336-6)
Supplement: Supplementary file 1 — Deconvoluted electrospray mass spectra of J591c-scFv pre- and post-reduction and conjugation with THP-mal. Table S2. Ex vivo biodistribution data (%ID/g) for 68Ga-THP-mal-J591-scFv in mice bearing DU145 (PSMA-) and Du145-PSMA (PSMA+) (mean and standard deviation are shown; n = 4 in each group). Figure S1. Electrospray mass spectra of J591c-scFv pre-TCEP treatment (top, 27,923.2 corresponds to disulfide formed from J591c linked to cysteine via a disulfide bond), J591c-scFv post-TCEP treatment (middle, 27,803.9 corresponds to J591-scFv), and TCEP-treated J591-scFv after incubation with THP-mal (bottom, 28,723.7 corresponds to THP-mal-J591-scFv adduct). Figure S2. The effect of different molar excesses of TCEP on dimerisation of J591-scFv. Lanes: 1: molecular weight markers; 2: non-reduced protein; 3: reduced protein (NuPAGE reducing agent); 4: 0.4:1 M ratio TCEP to protein; 5: 1:1 M ratio; 6: 2:1 M ratio; 7: 5:1 M ratio; 8: 10:1 M ratio; 9: 15:1 M ratio; 10: 20:1 M ratio; 11: 30:1 M ratio. Figure S3. FPLC purification of THP-mal-J591-scFv conjugate on Superdex 75 HR 10/30 size-exclusion column eluted with phosphate-buffered saline at 0.5 ml/min. Conjugate elutes before free THP-mal. Free THP-mal can be seen eluting at 15–18 min, monomeric THP-conjugated protein at 10 min and dimeric protein at 8 min. Figure S4. Exemplar data showing radiolabelling efficiency (% protein bound, determined by ITLC) at different protein conjugate concentrations and times. At concentrations of 0.25 mg/ml or higher, labelling efficiency was 100% at all time points from 10 s onward (the first data point in each series represents a sample taken after 10 s incubation). Figure S5. SDS-PAGE analysis of serum stability of 68Ga-THP-mal-J591-scFv. A: radioactive gel analysed with cyclone phosphor imager. B: gel stained with Coomassie blue. Lanes: 1: molecular weight markers; 2: human serum incubated with ammonium acetate-buffered 68Ga eluate; 3: radiolabelled 68Ga-THP-mal-J591-scFv conjugate contro [file 13550_2017_336_MOESM1_ESM.pdf]

Supplementary data for:

Simple, mild, one-step labelling of proteins with ^{68}Ga using a tris(hydroxypyridinone) bifunctional chelator: ^{68}Ga-THP-scFv targeting the prostate specific membrane antigen

Saima Nawaz, Gregory E D Mullen, Kavitha Sunassee, Jayanta Bordoloi, Philip J Blower, and James R Ballinger

Methods

Protein purification and analysis

Size exclusion chromatography: Purification of J591c-scFv and its THP-mal conjugate was performed using FPLC size exclusion column chromatography using a Superdex 75 10/300 GL column (GE), eluting with phosphate buffered saline (pH 7) at a flow rate of 0.5 ml/min and maximum pressure of 1.8 MPa.

Polyacrylamide gel electrophoresis and Western blotting were used to evaluate purity of the size-exclusion-purified J591c-scFv, using pre-cast polyacrylamide gels (NuPAGE 12% Novex Bis-Tris mini gels with MES buffer, Life Technologies) and Western blotting. For the preparation of samples, 10 µl aliquots of J591-scFv were placed in microcentrifuge tubes and 5 µl of NuPAGE LDS loading buffer (4x) was added. For reduced samples, the reducing agent (NuPAGE reducing agent of strength 10x, 2 µl) was added along with NuPAGE LDS buffer of 4x concentration (5 µl). Samples were placed on a heating block for 5 min at 95°C and then cooled on ice for 1 min. The NuPAGE chambers were filled with the MES buffer (50 ml of 20x MES buffer + 950 ml of deionised water). Novex sharp pre-stained protein standard was loaded in one of the wells. The gel was run for 40 min at a nominal voltage of 200 V with constant current of 125 mA, developed with SimplyBlue SafeStain (Thermo Fisher Scientific) for 2 h and destained with water overnight. In the case of radiolabelled proteins, before staining, activity markers were placed on the gels which were then imaged with a phosphorimager.

For Western blotting, the 12% gel was prepared as above; instead of staining, the gel was loaded on a nitrocellulose transfer membrane and developed in non-reducing transfer buffer (NuPAGE transfer buffer (20x) 50 ml + methanol (100 ml) + deionised water (850 ml)) for 1 h at constant 30 V and current of 170-130 mA. After 1 h the transfer membrane was

removed and placed in a blocking agent (4% milk in 50 ml of PBS with 0.05% Tween 20) for 90 min, washed briefly with PBS, then placed in a solution containing antibody against His-tag (6.3 µl of antibody diluted (1:4000 dilution) by adding 25 ml of PBS with 0.5% BSA, which is 0.125 g/25 ml of BSA) overnight at 4°C with shaking. The transfer membrane was washed four times with 10 ml of PBST (1 l of phosphate buffered saline with 0.05% Tween 20) before incubating with secondary antibody (goat anti-mouse horseradish peroxidase) for 60 min (1:5000 dilution, approximately 4 µl of HRP in 20,000 µl of PBS). The transfer membrane was washed with PBST four times before immersing it in the solution of substrate DAB [(3,3-diaminobenzidine) Peroxidase Substrate Tablet Set from Sigma Aldrich] (one DAB tablet and one Urea Hydrogen Peroxide tablet dissolved in 15 ml of ultrapure water). The transfer membrane was developed in the dark until the brown–black precipitation was visible.

Instant thin layer radiochromatography (ITLC)

Protein labelling with gallium-68 was assessed by instant thin layer chromatography (ITLC) using ITLC-SA (Agilent) developed with 0.1 M sodium citrate buffer (pH 6) as the mobile phase. Under these conditions unbound gallium-68 moved with the solvent front (Rf = 1) and radiolabelled conjugated protein remained at the origin (Rf = 0).

High performance radiochromatography

The radiolabelled protein conjugates were analysed using a size-exclusion column (SEC-2000), sample volume 20 µl, eluting with phosphate buffered saline (pH 7), flow rate 1 ml/min, UV detection at 280 nm and gamma detection with a sodium iodide detector.

Serum stability

THP-J591c-scFv (200 µl, 0.4 µg/µl) was radiolabelled by incubation with 300 µl of ^{68}Ga generator eluate (60 MBq) for 20 min at room temperature and labelling efficiency found to be >99% by ITLC. Without further purification or modification, the radiolabelled conjugated

J591c (100 µl) was mixed with serum (200 µl) and physiological saline (100 µl). The mixture was incubated at 37°C and samples (5 µl each) were collected at 30, 60, 120, 180, 240 and 300 min and stored at -80°C until all samples had been collected. The behaviour of unchelated Ga-68 in serum was determined by incubating 10 µl of ammonium acetate buffered ^{68}Ga generator eluate with serum as described above, incubating for up to 60 min at 37°C, taking 5 µl samples and storing them at -80°C until all the time points were collected for gel electrophoresis analysis. ^{68}Ga-THP-mal-J591c-scFv incubated in saline and sampled similarly was used as a reference. For analysis by SDS-PAGE, the samples (5 µl) were mixed with 10 µl of LDS buffer (lithium dodecyl sulfate, pH 8.4) and applied on the gel (10 µl). The gel was developed in MES buffer (2-ethanesulfonic acid) for 40 min at 200 V and 120-130 A. Afterwards the gel was removed, activity markers were placed and it was analysed by phosphorimager and then stained with Coomassie blue.

PET imaging and quantification

Dynamic imaging (n = 1 each group) was performed over the time period of 3 h from the time of injection, 25 days after tumour inoculation, using a BioScan nanoPET-CT PLUS (Mediso) scanner using their proprietary acquisition software (Nucline, version 2.00). CT was performed with an x-ray tube voltage of 45 kVp, 600 ms of exposure time, and 360 projections. This scan took 10 min to obtain. Dynamic PET scans were acquired within a 94.7-mm field of view from 0 to 190 min after tail vein injection of the tracer. Acquisition took place in 1–5 coincidence mode with a coincidence window of 5 ns and a 400- to 600-keV energy window. The dynamic PET data were reconstructed using Nucline software (version 2.00). Images were constructed based on 0.4 mm^3 voxels for PET and 0.21 mm^3 for CT. Image processing and analysis were performed using Vivoquant software (version 1.23). Before analysis, both CT and PET images were realigned and processed to a voxel size of

0.21 mm^3 and the PET output calibrated to display MBq per voxel. Regions of interest (ROIs) for each data file were produced using freehand segmentation. SUV_{max} values were obtained from regions of interest near the centre of each selected organ/tumour, using the total activity within the image (excluding activity within the tail) as the total injected dose. Time–activity curves were produced from 15-min bins.

Results

Supplementary Table S1. Deconvoluted electrospray mass spectra of J591c-scFv pre- and post-reduction and conjugation with THP-mal.

Sample	Deconvoluted mass	Assignment
J591c-scFv pre-TCEP treatment	27923	J591c-scFv cysteine disulfide conjugate
J591c-scFv post-TCEP treatment	27804	J591c-scFv free thiol form
THP-mal-J591c-scFv	28724	THP-mal-J591c-scFv
THP-mal	920.4	THP-mal

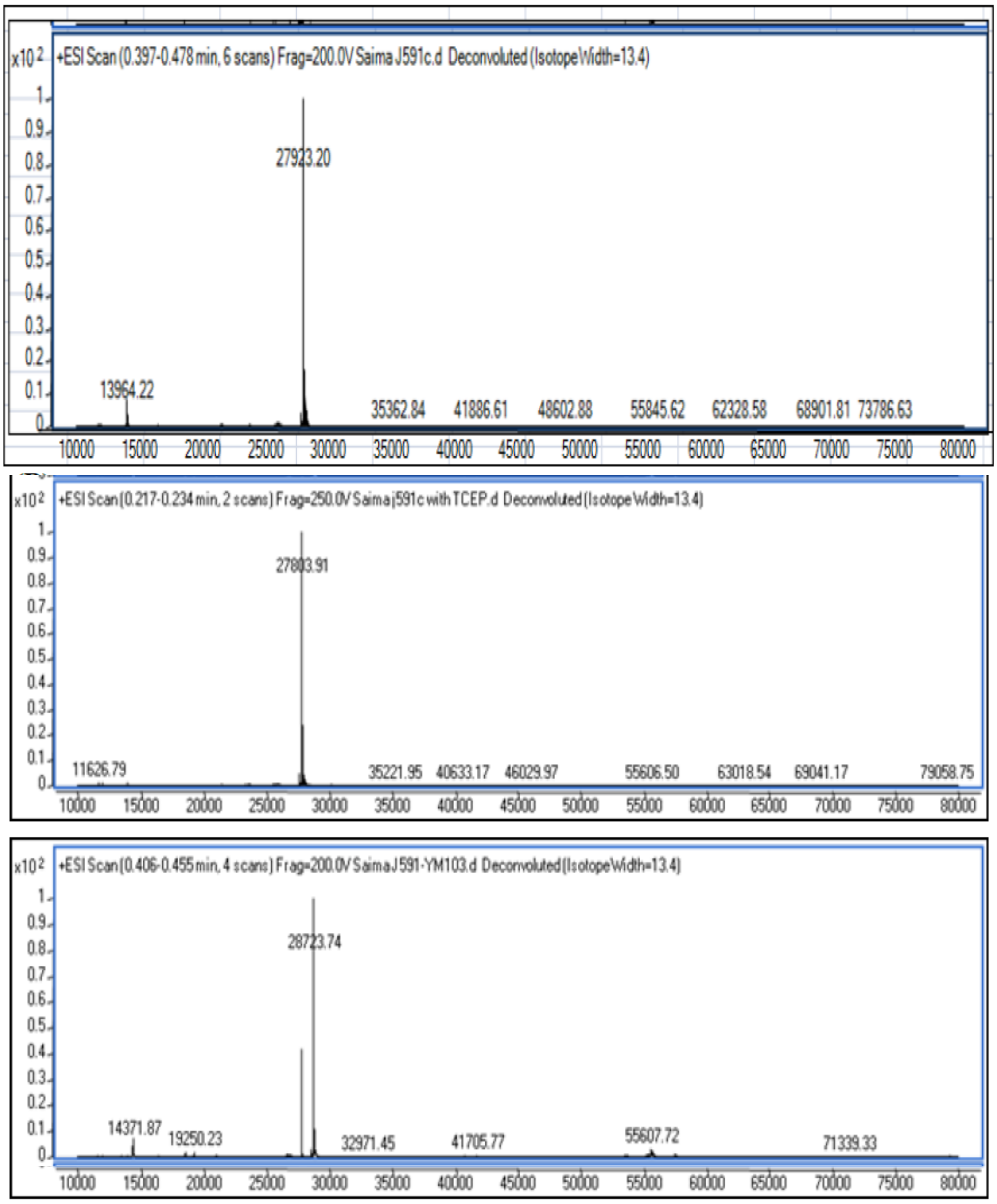

Supplementary Figure S1. Electrospray mass spectra of J591c-scFv pre-TCEP treatment (top, 27923.2 corresponds to disulfide formed from J591c linked to cysteine via a disulfide bond); J591c-scFv post-TCEP treatment (middle, 27803.9 corresponds to J591-scFv), and TCEP-treated J591-scFv after incubation with THP-mal (bottom, 28723.7 corresponds to THP-mal-J591-scFv adduct).

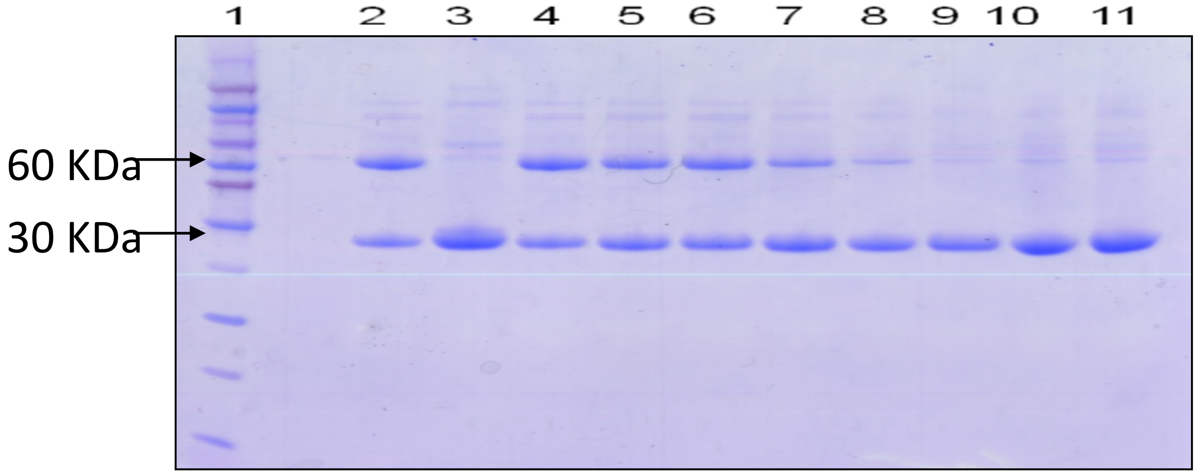

Supplementary Figure S2. The effect of different molar excesses of TCEP on dimerisation of J591-scFv. Lanes: 1: molecular weight markers; 2: non-reduced protein; 3: reduced protein (NuPAGE reducing agent); 4: 0.4:1 molar ratio TCEP:protein; 5: 1:1 molar ratio; 6: 2:1 molar ratio; 7: 5:1 molar ratio; 8: 10:1 molar ratio; 9: 15:1 molar ratio; 10: 20:1 molar ratio; 11: 30:1 molar ratio.

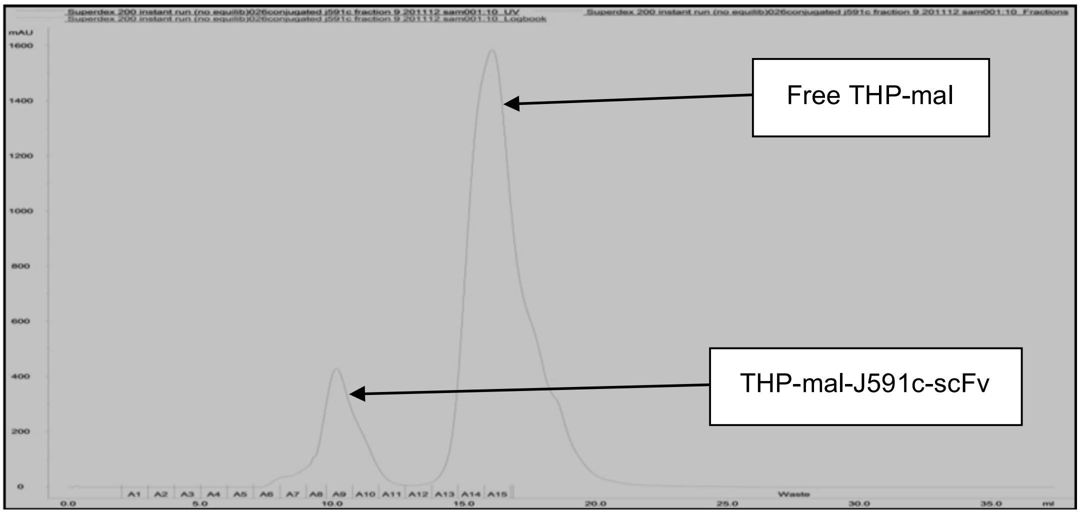

Supplementary Figure S3. FPLC purification of THP-mal-J591-scFv conjugate on Superdex 75 HR 10/30 size exclusion column eluted with phosphate-buffered saline at 0.5 ml/min. Conjugate elutes before free THP-mal. Free THP-mal can be seen eluting at 15-18 min, monomeric THP-conjugated protein at 10 min, and dimeric protein at 8 min.

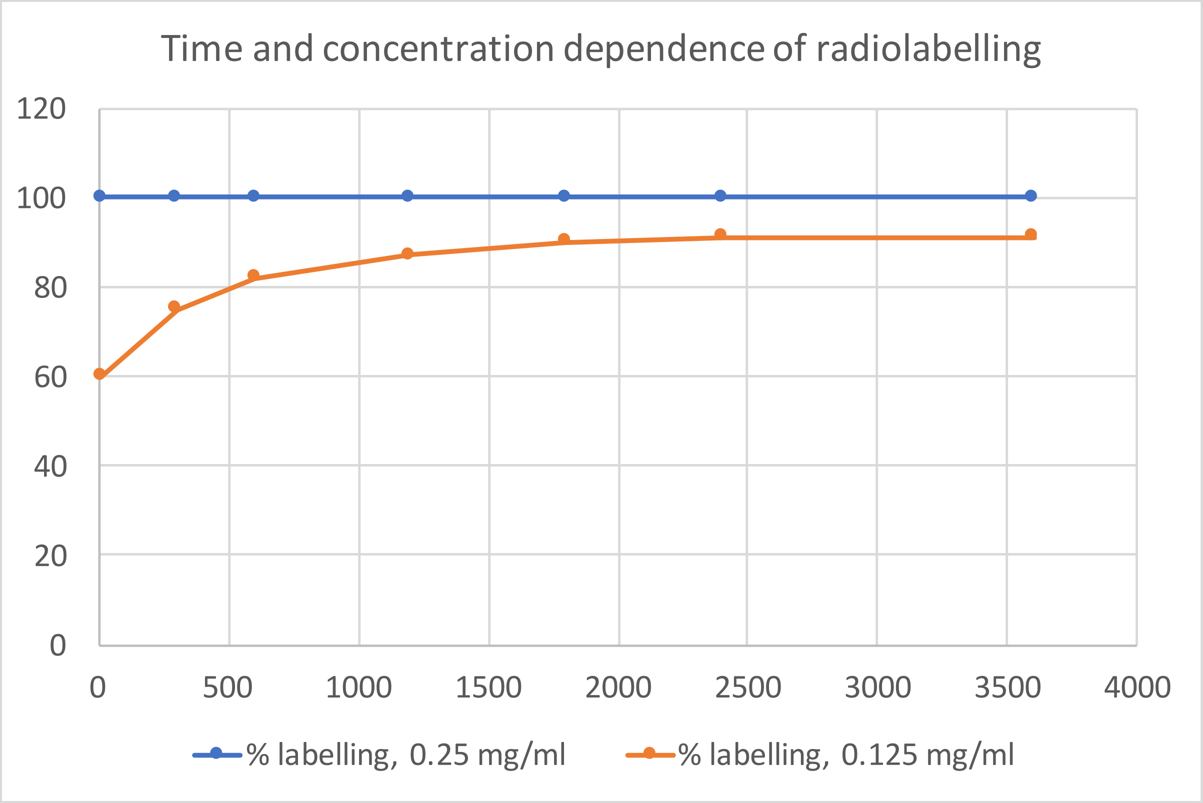

Supplementary Figure S4. Exemplar data showing radiolabelling efficiency (% protein bound, determined by ITLC) at different protein conjugate concentrations and times. At concentrations of 0.25 mg/ml or higher, labelling efficiency was 100% at all time points from 10 s onward (the first data point in each series represents a sample taken after 10 s incubation).

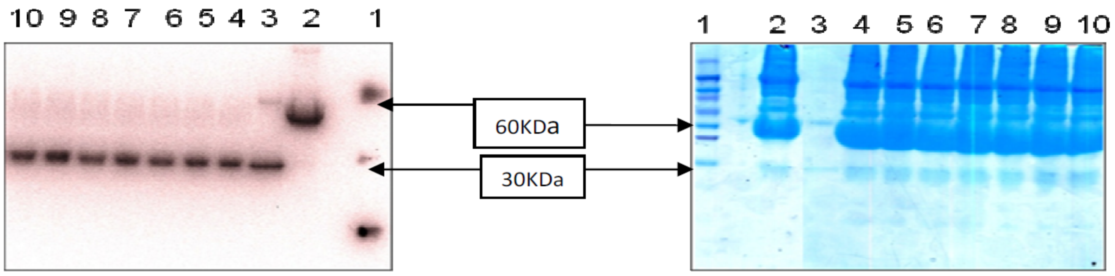

Supplementary Figure S5. SDS-PAGE analysis of serum stability of ^{68}Ga-THP-mal-J591-scFv. A: radioactive gel analysed with Cyclone phosphor imager. B: gel stained with Coomassie blue. Lanes: 1: molecular weight markers; 2: human serum incubated with ammonium acetate buffered ^{68}Ga eluate; 3: Radiolabelled ^{68}Ga-THP-mal-J591-scFv conjugate control (without serum incubation); 4: conjugate incubated with human serum for 1 min; 5: 30 min; 6: 60 min; 7: 120 min; 8: 180 min; 9: 240 min; 10: 360 min.

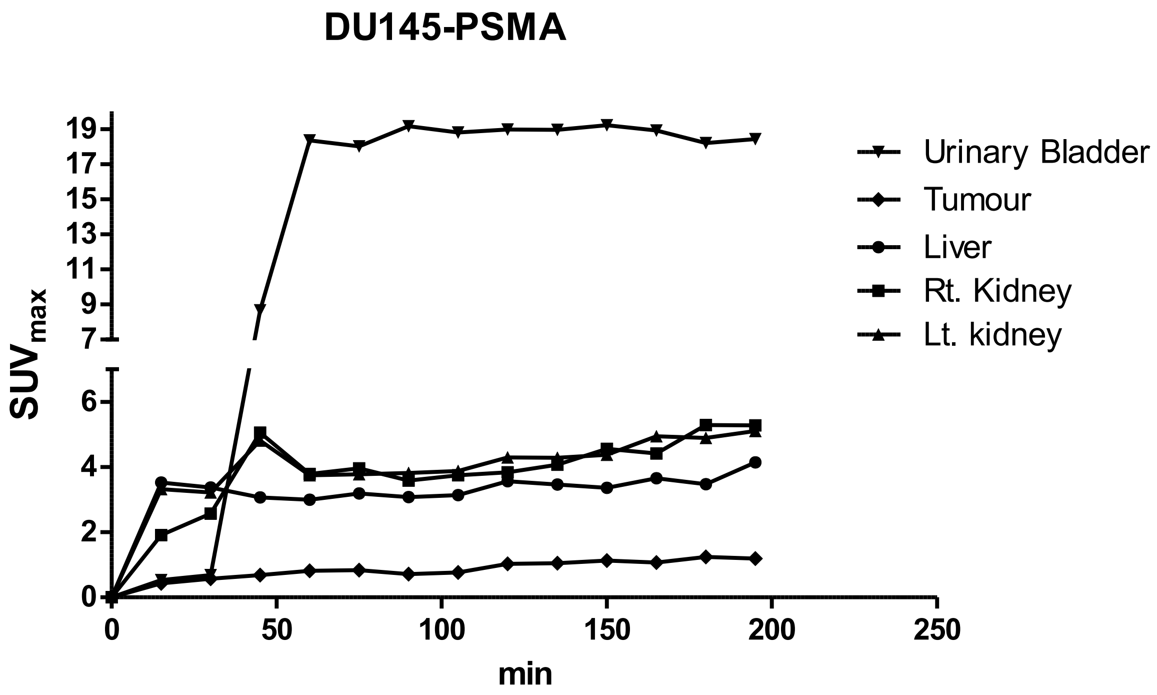

Supplementary Figure S6. Time course of ^{68}Ga-THP-mal-J591c-scFv activity (SUV_{max}) in DU145-PSMA xenografts derived from serial images in a single mouse determined by PET imaging. A region of interest near the centre of each organ was drawn from which SUV_{max} was determined in each 15 min bin.

organ	PSMA- ave	PSMA- SD	PSMA+ ave	PSMA+ SD
blood	1.3	0.2	0.8	0.4
tumour	0.5	0.2	5.4	0.5
stomach	0.2	0.1	0.5	0.3
small intestine	0.4	0.1	0.6	0.2
large intestine	0.3	0.2	0.8	0.2
spleen	3.0	1.4	3.4	0.7
liver	3.4	1.5	4.9	1.1
kidneys	41.5	12.7	89.3	10.0
heart	0.6	0.1	1.5	0.3
lungs	3.4	1.9	4.4	1.5
trachea&thyroid	0.7	0.3	1.6	0.5
salivary gland	0.4	0.2	0.9	0.2
muscles	0.2	0.1	0.4	0.2
bone	0.3	0.1	1.7	2.0
reproductive organ	0.4	0.2	0.7	0.2
tail	11.4	13.6	6.9	9.8
skin&fur	0.6	0.3	0.8	0.2

Supplementary Table S2. *Ex vivo* biodistribution data (%ID/g) for ^{68}Ga-THP-mal-J591-scFv in mice bearing DU145 (PSMA-) and Du145-PSMA (PSMA+) (mean and standard deviation are shown; n = 4 in each group).

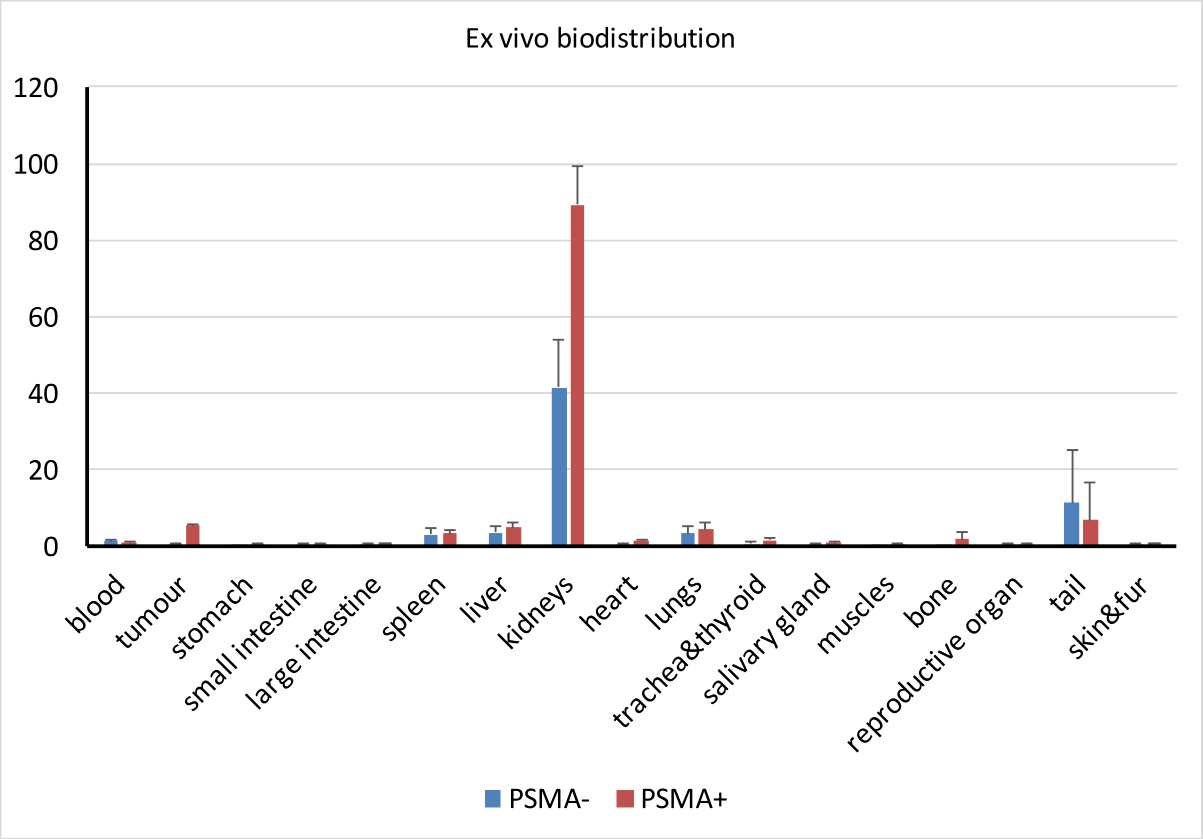

Supplementary Figure S7. *Ex vivo* biodistribution data for ^{68}Ga-THP-mal-J591-scFv in mice bearing DU145 (PSMA-, blue) and DU145-PSMA (PSMA+, red), 90 min post-injection. Error bars represent standard deviation (n = 4 per group). Data are the same as those shown in the main manuscript (Fig. 5) but expanded to include kidneys.
